# Detecting the start of an influenza outbreak using exponentially weighted moving average charts

**DOI:** 10.1186/1472-6947-10-37

**Published:** 2010-06-29

**Authors:** Stefan H Steiner, Kristina Grant, Michael Coory, Heath A Kelly

**Affiliations:** 1 Dept. of Statistics, University of Waterloo, Waterloo, Ontario Canada; 2 Epidemiology Unit, Victorian Infectious Diseases Reference Laboratory, Australia; 3 Queensland Health and University of Queensland, Australia

## Abstract

**Background:**

Influenza viruses cause seasonal outbreaks in temperate climates, usually during winter and early spring, and are endemic in tropical climates. The severity and length of influenza outbreaks vary from year to year. Quick and reliable detection of the start of an outbreak is needed to promote public health measures.

**Methods:**

We propose the use of an exponentially weighted moving average (EWMA) control chart of laboratory confirmed influenza counts to detect the start and end of influenza outbreaks.

**Results:**

The chart is shown to provide timely signals in an example application with seven years of data from Victoria, Australia.

**Conclusions:**

The EWMA control chart could be applied in other applications to quickly detect influenza outbreaks.

## Background

Influenza viruses cause seasonal outbreaks in temperate climates, usually during winter and early spring, and are endemic in tropical climates. The severity and length of influenza outbreaks vary from year to year. Quick and reliable detection of the start of an outbreak is needed for a number of reasons. Reminders can be made for eligible persons to be vaccinated. Once the influenza season has commenced, hospitals may wish to change admission procedures, depending on the anticipated number of patients with an influenza-like illness (ILI) requiring hospitalisation. For instance, hospitals might decide to reduce bookings for elective surgery in anticipation of increased acute admissions for influenza and its complications. Also, at relatively higher levels of ILI activity, wards admitting patients who are immunosuppressed may elect to roster only staff who have been vaccinated against influenza in order to protect those highly susceptible patients.

Recognising the influenza season is also important for modellers who attempt to estimate excess influenza-associated morbidity and mortality. Models require independent verification of the weeks during which influenza circulation exceeded a nominal baseline level [1]. Estimation of influenza vaccine effectiveness also requires definition of the influenza season, since influenza vaccine should only prevent influenza when the virus is circulating [2].

## Methods

### Literature review

Detecting changes in influenza activity over time has direct parallels in industrial applications where the use of control charts to monitor a time series for changes in baseline activity has a long history [3].

Reviews of the use of control charts for the prompt detection of outbreaks include those by Woodall [4] and Tsui et al. [5].

The simplest approach for detecting deviation from the baseline is based on the classical Shewhart type chart [3]. With a Shewhart chart, decisions regarding whether or not to signal an outbreak depend only on the observed measure of influenza activity (raw or residual) from the current time period. Serfling [6] suggested monitoring weekly observed minus expected influenza deaths, where the expected deaths were predicted using a time series regression model fit to historical data. More recently, Hashimoto et al. [7] suggested a Shewhart chart based on weekly ILI data from sentinel medical institutions and Viboud et al. [8] and Anderson et al. [9] extended the Serfling approach to monitor weekly ILI data from sentinel GPs where expected counts are based on a model fit to historical data that best matches the recent pattern.

Using observed minus expected counts [6,8,9] rather than actual counts, changes the implicit goal of the monitoring. Large observed minus expected counts (model residuals) suggests behaviour different than what we expected. For influenza we expect relatively large increases in activity that will be sustained over a number of weeks or months. In temperate climates we also expect a strong seasonal pattern with increased activity during the winter. As such, seasonal outbreaks may not correspond to large residuals, given that the expected counts would be high. However, our goal is the detection of an influenza outbreak, whether it corresponds to the expected seasonal activity or otherwise.

Cumulative Sum (CUSUM) charts are sequential monitoring methods where the current magnitude of the chart statistic, and thus the decision as to whether or not the chart should signal, depends on the observed (and possibility expected) counts from a number of recent time periods rather than a single time period as with a Shewhart chart. In the influenza monitoring context, a CUSUM chart was proposed by Muscatello et al. [10] for monitoring emergency department observed ILI counts minus the count from seven days prior. Thresholds are set heuristically based on a best fit of historical data. This approach is effective for detecting short term changes in influenza activity; however unfortunately the CUSUM has no intuitive interpretation.

From the industrial process monitoring literature [3] we know that Shewhart charts are good at detecting sudden large process changes, while sequential methods, such as CUSUM charts, are better for smaller sustained or gradual changes. As influenza outbreaks typically result in a large change in observed activity we may conclude that Shewhart methods would be ideal. However, at the start of an outbreak there is a transition period where activity is increasing, so the change from baseline activity to an established outbreak is not instantaneous. Also, there can be considerable variation, due to small numbers of counts and mild self limiting (unimportant) outbreaks, in observed activity even when there is no defined outbreak. As such it is not immediately clear whether a Shewhart or CUSUM approach is preferred. Cowling et al. [11] compare a variety of methods including time series methods, regression and CUSUM. However, there are many variations on the approaches, and, as discussed earlier, methods based on model residuals have a different goal than methods based on raw counts.

As a compromise between Shewhart and sequential approaches, such as CUSUM, we can modify the Shewhart approach by including runs rules which increases the sensitivity to small sustained changes. Runs rules can take many forms [3]. In the context of monitoring influenza, Toubiana et al. [12] and Watts et al. [13] discuss the use in France and Australia respectively of an ILI monitoring approach based on sentinel GPs that signals only when the observed count is above a threshold for two consecutive weeks. A similar idea comes from Muscatello et al. [14] who propose basing signals on a four week moving average.

### Data source

Our study was based in Victoria, the second most populous state of Australia with an estimated population of 5.2 million people. We monitored the level of influenza activity in the community using weekly laboratory confirmed influenza notification data. Laboratory confirmed influenza is a notifiable disease in Victoria and it is a legal requirement that cases are notified in writing by the responsible laboratory and medical practitioner within five days of diagnosis to the Victorian Government Department of Health [15]. The number of laboratory confirmed influenza diagnoses depends on the prevalence of influenza and testing behaviour of clinicians responsible for the diagnosis and management of influenza. We have assumed that testing out-of-season (December - April in the southern hemisphere) would be roughly constant while, during the influenza season and during an outbreak of sporadic influenza, testing will increase.

**Figure 1:**
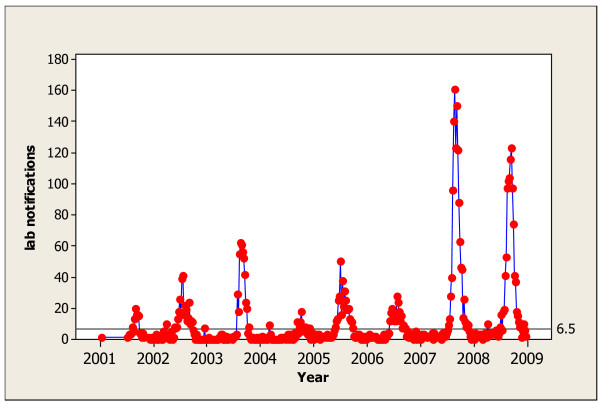
**Victorian Weekly Laboratory Notifications of Influenza 2002-2008 With Shewhart Chart Threshold of 6.5**.

All laboratory tests were conducted at the Victorian Infectious Diseases Reference Laboratory (VIDRL) from patients with ILI from sentinel general practices who were tested for influenza and from patients tested as part of routine clinical mangement. Sentinel general practices are community-based practices that provide surveillance data on infectious diseases. It is generally assumed that sentinel practices represent all community practices and information from those practices describes infectious disease activity in the community [16]. Laboratory testing used polymerase chain reaction (PCR) assays for the diagnosis of influenza [17]. The weekly VIDRL laboratory confirmed influenza counts for the period 2002-2008 are shown in Figure 1. The seasonal influenza outbreaks are clearly visible. The start of each influenza season corresponds to a rapid increase in the number of laboratory notifications. It therefore seems reasonable the start of an influenza outbreak should be relatively easy to detect prospectively.

We have used laboratory confirmed influenza as a specific outcome in this study. We have previously shown that, although the syndrome of ILI corresponds to influenza detections in our laboratory [13], only about 40% of all ILI diagnoses by sentinel general practitioners in Victoria between 2003-7 were confirmed as influenza [2]. The median interval between symptom onset and registration for a laboratory test was three days for a patient recruited through sentinel GPs in Victoria in 2007 and 2008. Testing is generally accomplished within 48 hours and results are automatically notified to the Health Department. The delay between recording of an episode of ILI at a sentinel general practice and confirmation of that ILI as being due to influenza would be generally less than one week.

### Establishing a threshold

Using a threshold on the observed weekly number of positive laboratory notifications corresponds to a Shewhart chart and is the simplest approach. We illustrate the difficulties with this approach in Figure 1 using a threshold of 6.5 positive notifications per week, a value chosen by inspection to detect the start of a season without signalling often out-of-season. Since we are using count data, any value between 6 and 7 will represent the same threshold. Using the empirical baseline data (i.e. when the process is assumed to be in-control) the false alarm rate with a threshold of 6.5 is 5/156 = 0.032. Due to the occasional isolated outliers from large counts out-of-season it is difficult to detect the start of the influenza season while avoiding frequent false alarms. False alarms occur when the monitoring procedure signals the start of an outbreak, but the increase in laboratory notifications is not sustained over a number of weeks.

One way to alleviate this problem of frequent false alarms is to base the detection on a smoothed version of the laboratory notification time series. This can be done several ways, for example Muscatello et al. [14] recommended using a four week moving average of laboratory notifications for monitoring. Their approach signals the start of the influenza season whenever the four-week moving exceeds a preset threshold. While effective, this approach has the disadvantage that there is an arbitrary sudden cutoff for those observations included in the smoother. We compare the performance of this MA(4) approach to the proposed EWMA method later in this paper.

### The exponentially weighted moving average (EWMA) control chart

To detect the start (and end) of an influenza outbreak we propose the exponentially weighted moving average (EWMA) control chart [18] defined as:(1)

where *y*_*t *_equals the number of laboratory notifications in week *t*, 0 < λ ≤ 1 and *E*_0 _= 0 (or some other suitable starting value). The EWMA "signals" the first time *E*_*t *_> *h*. Note that applying the EWMA formula (1) recursively we get *E*_*t *_= λ *y*_*t *_+ λ (1-λ)*y*_*t*-1 _+ λ (1-λ)^2 ^*y*_*t*-2 _+ ... In other words, as the name suggests, the EWMA statistic *E*_*t *_is a weighted average of all previous observed *y*_*t *_values with weights that become (exponentially) smaller as we go further back in time. As such the EWMA statistic provides a local estimate of mean level of the process that produces the observed *y*_*t *_values. Thus, unlike the CUSUM [18] the EWMA statistic provides a ready simple interpretation. EWMA charts have previously been proposed for monitoring community based epidemics as part of the ESSENCE surveillance system [19]. The ESSENCE system is based on non traditional and syndromic information and has a much wider scope, i.e. detection of not just influenza outbreaks, and uses very different data than our proposal.

**Figure 2:**
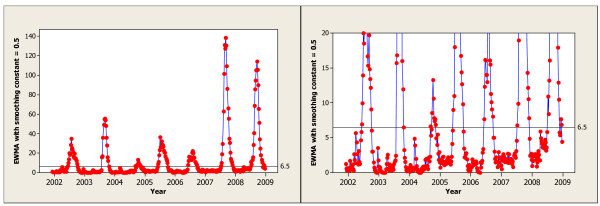
**EWMA with *λ *= 0.5 Applied to Victoria Laboratory Notification Data**.

To apply the EWMA chart we need to choose the smoothing constant, *λ*, and threshold, *h*. Due to the usually rapid increase and decrease in the number of laboratory notifications of influenza we would want only a little smoothing. Thus, we would choose a fairly large value for *λ *like 0.5. Figure 2 shows the EWMA chart for the Victorian laboratory notification data with *λ *= 0.5 (both left and right panels show the same chart, the right panel restricts the range of the vertical axis). This value for *λ *is larger than that used in most industrial applications were the goal is to detect more gradual sustained changes. Note that with *λ *= 1, the EWMA control chart simplifies to the Shewhart control chart [3] as shown in Figure 1.

The choice of threshold, *h*, requires a tradeoff between protection from false alarms and the ability to detect real changes quickly. Examining the EWMA of the historical data, shown in Figure 2, suggests a threshold between 6 and 7 is a reasonable compromise. We added a threshold of 6.5 to the EWMA in Figure 2 for illustration. In the next subsection we examine the nature of the tradeoff in more detail.

The proposed influenza detection procedure is based on whether the EWMA statistic *E*_*t*_, as given in (1), is above or below the threshold. While *E*_*t *_> *h *there is evidence of increased influenza activity. We define the first time in the year when the EWMA is above threshold as the start of influenza season. To meet our secondary goal, we will signal the end of the influenza outbreak (or season) as the first time after the start of the outbreak that the EWMA falls below the threshold. Thus, mathematically, if *E*_*t *_> *h *and *E*_*t*-1 _<*h*, i.e. the EWMA signals the start of an influenza outbreak at time *t*, the EWMA signals the end of that outbreak at time *s *> *t*, where *E*_*s *_<*h *and *E*_*t*+1_, *E*_*t*+2_, ..., *E*_*t*+*s*-1 _> *h*.

### Quantifying the performance of the EWMA control chart using the average run length

We plan to apply the EWMA prospectively to new laboratory notification data. If we assume the past data are representative of the type of data we will see in the future, we can use the historical data to set the threshold and assess the likely performance of the EWMA control chart. To quantify the performance we use the average run length (ARL), that is, the average number of weeks until a signal [18]. It is not appropriate to use false alarm rates or power to characterize the performance of a sequential control chart like an EWMA. Even with no change in activity, the chance of a signal at time *t *is not constant as it depends on the level of the EWMA at time *t*-1. We want a long ARL when there is only baseline influenza activity, while a good monitoring procedure will have a short ARL during an influenza outbreak.

## Results and Discussion

**Figure 3:**
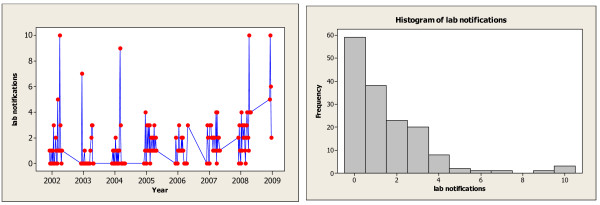
**Histogram and Time Series Plot of the Laboratory Notifications in the Baseline Period**.

To apply the EWMA control chart to the Victoria influenza data we first address the question of the ARL to a false alarm, called the in-control ARL. In the historical data there were no large outbreaks in the (Southern Hemisphere) summer months. We use the five months December to April inclusive to define a period where there is only baseline influenza activity. Changes in laboratory confirmed influenza counts from the baseline rate represent outbreaks. The two plots in Figure 3 summarize the available historical baseline activity data. The plot in the left panel shows the notifications over time, while the right panel summarizes the same data in a histogram.

We have a total of 156 observations for the baseline weekly laboratory notifications. The number of positive influenza tests in the baseline period, as shown in the right panel of Figure 3, is low, averaging just 1.5 per week. Also, the pattern over time is fairly constant and autocorrelations are small. Thus it is reasonable to assume independence across weeks in the baseline period. However, finding a parametric distribution that fits the observed histogram in Figure 3 proved difficult. The natural choice of a Poisson distribution fit poorly due mostly to the over-dispersion represented by the observed counts of 9 and 10 as seen in the right panel of Figure 3. Instead we proceeded with the empirical distribution.

**Figure 4:**
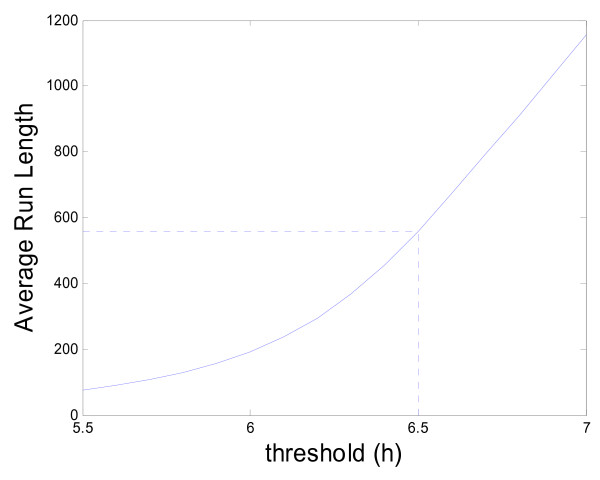
**Plot of the Baseline Average Run Length (ARL) by the threshold *h***.

We use a Markov chain to approximate the steady state baseline ARLs with different thresholds [20]. The results are given in Figure 4. With the previously selected threshold of 6.5, we obtain a (cyclical) steady state [18] average run length of 556 weeks. This means that using the proposed EWMA, we expect, on average, just one out-of-season false alarm roughly every 25 years if the influenza activity remains at the baseline level (recall there are only 5 out-of-season months every year).

Next, we consider the speed with which the EWMA approach will signal changes in influenza activity from the baseline rate. Here we need an assumption for the distribution of the additional laboratory notifications due to the outbreak. We apply the following simple model: *Y*_*t *_= *B*_*t *_+*O*_*t*_, where *Y*_*t *_is a random variable whose realizations give the observed laboratory notifications, *B*_*t *_is a random variable for the baseline influenza activity whose distribution is given by the empirical distribution shown in the histogram in Figure 3, and *O*_*t *_is a random variable that represents the additional laboratory notifications due to the influenza outbreak. We assume *O*_*t *_has a Poisson distribution with mean *μ*. As the mean *μ *increases the severity of the influenza outbreak increases and with mean zero we have only baseline activity.

**Figure 5:**
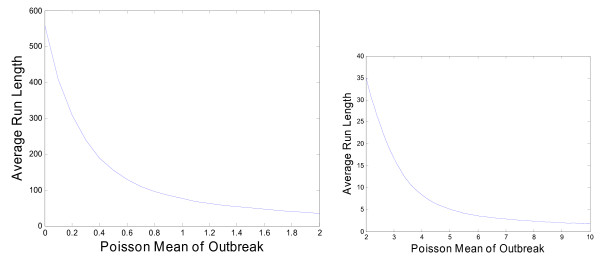
**Plot of the Average Run Length (ARL) in weeks by the Size of the Outbreak**.

Figure 5 shows how the EWMA ARL changes with *μ*. The EWMA very rapidly detects any outbreak with Poisson mean greater than about 6. Given the size of the outbreaks shown in Figure 1, we expect the EWMA chart to detect the typical seasonal influenza outbreak within one or two weeks of the outbreak's start. Note, however, that this analysis is only designed to give some indication of performance. To determine the ARLs we assumed a step change in the Poisson mean reflected the start of an influenza outbreak. In reality, an influenza outbreak is likely sudden but not instantaneous. Also, this analysis assumes outbreaks continue indefinitely at a constant rate. Smaller outbreaks may not be detected before they subside. However, our main goal is detecting large influenza outbreaks.

### Comparison of Methods

**Figure 6:**
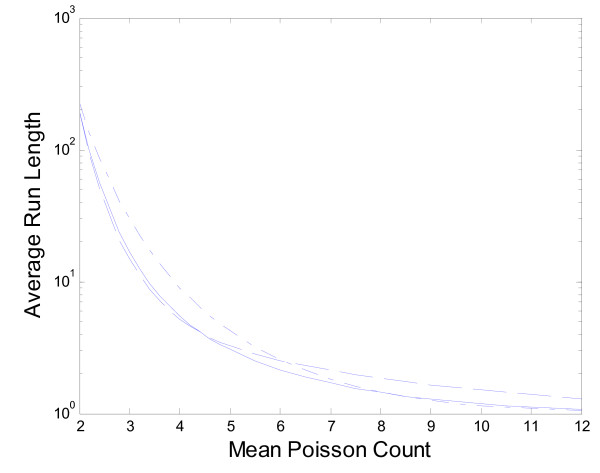
**Comparison of ARL for EWMA, MA(4) and Shewhart Methods Solid line: EWMA, dashed line: MA(4), dashed dot line: Shewhart**.

Next we compare the proposed EWMA method using *λ *= 0.5 with the four period moving average, MA(4), an approach advocated by Muscaltello et al. [14] and the Shewhart approach where we simply compare the observed count each week with a threshold. In this comparison we assume the number of ILI cases out-of-season follows a Poisson distribution with mean 2 and model outbreaks of various sizes by increasing the Poisson mean. We do not use the empirical out-of-season Victoria data here because, due to the small amount of data, it is not possible to find a Shewhart chart with a reasonably large in-control (or out-of-season) ARL. By setting up a Markov chain model that takes into account all four values that make up the moving average and because each count is an integer we can determine exact results for the performance of the MA(4) method. The results for the Shewhart chart are also exact while for the EWMA we use the Markov chain approximation. Figure 6 gives the results on a log scale for the average run lengths of the three approaches. We were unable to exactly match the in-control performance of the three charts because of the inherent discreteness of the count data. With control limits of 4.4, 3.9 and 6.9 for the EWMA(*λ *= 0.5), MA(4) and Shewhart approaches respectively we have a steady state in-control ARL of 190 for the EWMA and MA(4) methods but 220 for the Shewhart approach. We see in Figure 6 that, as expected, the EWMA and MA(4) approaches are quicker to detect outbreaks than the Shewhart approach when the outbreak is relatively small. In addition for very large shifts the Shewhart chart is marginally better than the EWMA approach while the MA(4) approach takes longer to signal. This comparison is limited for our context because with influenza outbreaks we expect sudden but not instantaneous large shifts in the mean number of counts. Modeling a more realistic influenza outbreak would require additional assumptions about how quickly changes take place and require either simulation or a much more complicated analysis to generate results. We feel that because the EWMA approach works very well in comparison to the MA(4) and Shewhart approaches for shifts of any size it is the preferred approach. Note in particular the EWMA is substantially better than the MA(4) approach for the larger shifts we hope to be able to detect quickly.

### Application of the EWMA chart

We applied the proposed EWMA control chart to the laboratory notification data from Victoria. The resulting EWMA chart is given in Figure 2. From Figure 2, the decision rules described earlier and the detailed records, we determined the signaled start and end weeks for the seasonal influenza outbreaks (see Table 1). Note that this determination was done in a prospective manner, that is, decisions were made at week *t *without looking at *y*_*t*+1_, *y*_*t*+2_, etc. For comparison we also included in Table 1 the signal dates as determined by a retrospective inspection of notification data by epidemiologists. The start and end of the influenza season was fairly clear for all years except 2004 when there was very mild seasonal influenza activity.

**Table 1:** Start and End Weeks of Victorian Influenza Season as Determined by Proposed EWMA Approach and Retrospective Analysis

			Retrospective Analysis			
			
EWMA (week #)			Start		End	
**Year**	**Start**	**End**	**Week #**	**Date**	**Week #**	**Date**

2002	24	41	23	June 3-9	41	Oct. 7-13
2003	31	43	31	July 28-Aug. 3	42	Oct. 13-19
2004	37	38	37	Sept. 6-12	46	Nov. 8-14
2005	23	40	22	May 30-June 5	40	Oct. 3-9
2006	23	39	23	June 5-11	38	Sept. 25-Oct. 1
2007	28	49	28	July 9-15	48	Nov. 26-Dec. 2
2008	28	48	28	July 7-13	48	Nov. 24-30

The EWMA and retrospective approaches differed by at most one week in detecting the start of the influenza season. The EWMA gave no false signals for the start of an influenza season. Similar results were obtained for the end of the season determination with the exception of 2004. In 2004 the EWMA was above the threshold for weeks 37 and 40 through 46. As such, the EWMA approach signaled the end of the influenza season in week 38, and the subsequent start of another outbreak in week 40 which ended in week 47. These two signaled outbreaks together closely match the results from the retrospective analysis.

We have illustrated the application of our proposed EWMA influenza monitoring procedure with data from Victoria. Applying the approach elsewhere should be straightforward. Given some years of historical data we could produce a plot like Figure 2 and use our judgment to select a reasonable threshold. The approach could also be used to monitor changes in other diseases. If detecting more moderate changes is the goal, smaller values of the smoothing constant *λ *would be preferred.

We have purposefully not used the seasonal nature of influenza to help us detect the start of an outbreak. Rather we use a local estimate of activity to determine if an outbreak has started. Using the seasonal time information is somewhat problematic since the start of the influenza season can vary considerable. Additionally, we required the monitoring procedure to be sensitive to any outbreak - not only the expected seasonal outbreak. As a result, the proposed EWMA procedure could also be useful for detecting influenza outbreaks in tropical climates where there is usually little or no seasonal effect.

We selected the EWMA threshold by applying the EWMA to some historical data and used our judgment to determine the best threshold. The threshold should be updated every few years to accommodate possible changes in the process such as changes in population, the number of tests conducted and the type of influenza tests commonly used.

It is questionable whether it is reasonable to incorporate a single measure capable of signaling the start of influenza outbreaks across large geographical areas such as Australia. A preferred approach would be to monitor influenza activity separately for smaller geographical areas such as states but this introduces other complications. With multiple EWMAs, the ARL to a false alarm is clearly smaller than that for each individual EWMA. Also for states with small population, the baseline number of laboratory notifications will be smaller and, relative to the mean, more variable than for larger states. For regions with larger populations and/or larger numbers of tests the thresholds would need to be higher, but the EWMA would still be appropriate. With larger counts the discreteness problem in selecting thresholds for the moving average and Shewhart approaches would be lessened.

## Conclusions

We propose a simple, robust method for detecting the start and end of the influenza season that can also rapidly detect "out of season" influenza outbreaks. The data used to determine the threshold at which an alert is signaled are readily available with minimal delay where laboratory confirmed influenza is a notifiable disease. The method we propose is simple to implement and the calculations are relatively simple to execute. Baseline data from historical non-influenza periods of several years should be used to select the threshold. This will balance the desire for few false alarms and quick detection of an outbreak and will also provide accurate indications of the numbers of cases and the rate of increased testing at the beginning of past influenza seasons.

The EWMA method can also be used in other surveillance programs for the rapid detection of other diseases. Moreover, since seasonality is not inherent in the application of the model, the method can be used in tropical climates where seasonality of disease may not be apparent.

## List of abbreviations

EWMA: exponentially weighted moving average; ILI: influenza-like illness; ED: emergency department; GP: general practitioners; CUSUM: cumulative sum; EARS: early aberration reporting system; VIDRL: Victorian Infectious Diseases Reference Laboratory; PCR: polymerase chain reaction assay; RSV: respiratory syncytial virus; ARL: average run length

## Competing interests

The authors declare that they have no competing interests.

## Authors' contributions

SS conceived the model, performed the analysis and drafted the manuscript. KAG provided all raw data for analysis, contributed to discussion about establishing a threshold and assisted with production of the manuscript. MC participated in discussion about refining the model and establishing a threshold and contributed to the analysis. HK conceived the study, participated in the study design, provided background information on influenza epidemiology and helped draft the manuscript.

All authors reviewed and approved the final draft of the manuscript.

## Pre-publication history

The pre-publication history for this paper can be accessed here:

http://www.biomedcentral.com/1472-6947/10/37/prepub

## References

[B1] Newall AT Wood JG Macintyre CR Influenza-related hospitalisation and death in Australians aged 50 years and older Vaccine 2008 26 17 2135 41 10.1016/j.vaccine.2008.01.051 18325639 PMC7125633

[B2] Kelly H Carville K Grant K Jacoby P Tran T Barr I Estimation of influenza vaccine effectiveness from routine surveillance data PLoS One 2009 4 3 e5079 10.1371/journal.pone.0005079 19333374 PMC2658741

[B3] Montgomery DC Introduction to Statistical Quality Control 2008 6 John Wiley and Sons, New York

[B4] Woodall WH The Use of Control Charts in Health-Care and Public-Health Surveillance (with discussion) Journal of Quality Technology 2006 38 89 134

[B5] Tsui K-L Chiu W Gierlich P Goldsman D Liu X Maschek T A review of healthcare, public health and syndromic surveillance Quality Engineering. 2008 online 1st October 2008 20 4 435 50

[B6] Serfling RE Methods for current statistical analysis of excess pneumonia-influenza deaths Public Health Rep 1963 78 6 494 506 19316455 PMC1915276

[B7] Hashimoto S Murakami Y Taniguchi K Nagai M Detection of epidemics in their early stage through infectious disease surveillance Int J Epidemiol 2000 29 5 905 10 10.1093/ije/29.5.905 11034976

[B8] Viboud C Boelle PY Carrat F Valleron AJ Flahault A Prediction of the spread of influenza epidemics by the method of analogues Am J Epidemiol 2003 158 10 996 1006 10.1093/aje/kwg239 14607808

[B9] Anderson E Bock D Frisen M Modeling influenza incidence for the purpose of on-line monitoring Statistical Methods in Medical Research 17 421 438 10.1177/0962280206078986 17698935

[B10] Muscatello DJ Churches T Kaldor J Zheng W Chiu C Correll P Jorm L An automated, broad-based, near real-time public health surveillance system using presentations to hospital Emergency Departments in New South Wales, Australia BMC Public Health 2005 22 5 141 15. 10.1186/1471-2458-5-141 PMC1361771 16372902

[B11] Cowling BJ Wong IO Ho LM Riley S Leung GM Methods for monitoring influenza surveillance data Int J Epidemiol 2006 35 5 1314 21 10.1093/ije/dyl162 16926216

[B12] Toubiana L Flahault A A space-time criterion for early detection of epidemics of influenza-like-illness Eur J Epidemiol 1998 14 5 465 70 10.1023/A:1007481929237 9744678

[B13] Watts CG Andrews RM Druce JD Kelly HA Establishing thresholds for influenza surveillance in Victoria Aust N Z J Public Health 2003 27 4 409 12 10.1111/j.1467-842X.2003.tb00418.x 14705303

[B14] Muscatello DJ Morton PM Evans I Gilmour R Prospective surveillance of excess mortality due to influenza in New South Wales Communicable Diseases Intelligence 2009 32 4 10.33321/cdi.2008.32.42 19374272

[B15] Department of Human Services Rural Infection Control Practice Group. Health (Infectious Disease) Regulations Melbourne RICPRAC 2001 Report No.: Policy no. 7.1 Contract No.: Document Number|.

[B16] Clothier H Turner J Hampson A Kelly H Geographic representativeness for sentinel influenza surveillance: implications for routine surveillance and pandemic preparedness Aust NZ J Public Health 2006 30 337 341 10.1111/j.1467-842X.2006.tb00846.x 16956163

[B17] Druce J Tran T Kelly H Kaye M Chibo D Kostecki R Laboratory diagnosis and surveillance of human respiratory viruses by PCR in Victoria, Australia, 2002-2003 J Med Virol 2005 75 1 122 9 10.1002/jmv.20246 15543580 PMC7166941

[B18] Lucas JM Saccucci MS Exponentially Weighted Moving Average Control Schemes: Properties and Enhancements Technometrics 1990 32 1 12 10.2307/1269835

[B19] Lombardo J Burhom H Elbert E Magruder S Lewis SH Loschen W Sari J Sniegoski C Wojcik R Pavlin J A systems overview of the Electronic Surveillance System for the Early Notification of Community-Based Epidemics (ESSENCE II) J Urban Health 2003 80 2 Suppl 1 i32 42 12791777 10.1007/PL00022313 PMC3456555

[B20] Steiner SH Grouped Data Exponentially Weighted Moving Average Control Charts Applied Statistics 1998 47 203-21612293397

